# Severe Pneumonia Caused by Influenza A (H1N1) Virus Successfully Managed with Extracorporeal Life Support in a Comorbid Former Preterm Infant

**DOI:** 10.3390/ijerph14040360

**Published:** 2017-03-31

**Authors:** Genny Raffaeli, Giacomo Cavallaro, Lorenza Pugni, Ernesto Leva, Andrea Artoni, Simona Neri, Chiara Baracetti, Mauro Cotza, Valerio Gentilino, Leonardo Terranova, Susanna Esposito, Fabio Mosca

**Affiliations:** 1 Neonatal Intensive Care Unit, Department of Clinical Sciences and Community Health, Fondazione IRCCS Ca’ Granda Ospedale Maggiore Policlinico, Università degli Studi di Milano, Via della Commenda 12, 20122 Milan, Italy; genny.raffaeli@unimi.it (G.R.); lorenza.pugni@mangiagalli.it (L.P.); chiara.baracetti@gmail.com (C.B.); fabio.mosca@mangiagalli.it (F.M.); 2 Department of Pediatric Surgery, Fondazione IRCCS Ca’ Granda Ospedale Maggiore Policlinico, Università degli Studi di Milano, Via della Commenda 12, 20122 Milan, Italy; ernesto.leva@policlinico.mi.it (E.L.); gentilinovalerio@hotmail.com (V.G.); 3 Angelo Bianchi Bonomi Hemophilia and Thrombosis Center, Fondazione IRCCS Ca’ Granda Ospedale Maggiore Policlinico, Università degli Studi di Milano, Via Pace 9, 20122 Milan, Italy; andrea.artoni@policlinico.mi.it; 4 Pediatric Anesthesiology and Intensive Care Unit, Department of Anesthesia and Critical Care, Fondazione IRCCS Ca’ Granda Ospedale Maggiore Policlinico, Via della Commenda 9, 20122 Milan, Italy; neri.simona@virgilio.it; 5 ECMO Team, Department of Cardiothoracic and Vascular Anesthesia and Intensive Care, IRCCS Policlinico San Donato, 20097 San Donato Milanese, Italy; mauro.cotza@grupposandonato.it; 6 Pediatric Highly Intensive Care Unit, Department of Pathophysiology and Transplantation, Fondazione IRCCS Ca’ Granda Ospedale Maggiore Policlinico, Università degli Studi di Milano, Via della Commenda 9, 20122 Milan, Italy; terranova.leonardo@gmail.com (L.T.); susanna.esposito@unimi.it (S.E.); 7 Pediatric Clinic, Department of Surgical and Biomedical Sciences, Università degli Studi di Perugia, Piazza Menghini 1, 06129 Perugia, Italy

**Keywords:** influenza A (H1N1) virus, pneumonia, extracorporeal life support (ECLS), neonate, preterm infant, young infant, neonatal intensive care unit, infection control measures, oseltamivir

## Abstract

Influenza A (H1N1) virus infection is a global health burden, leading to significant pediatric morbidity and mortality. Prematurity, young age and comorbidities are important risk factors for unfavorable outcomes. Preventive strategies, such as healthcare workers and household contacts vaccination as well as the implementation of infection control practices during the epidemic season, are crucial to protect the most vulnerable populations. Early diagnosis, timely administration of antiviral drugs and supportive therapy are crucial to lead to a complete recovery. When conventional treatment fails, extracorporeal life support (ECLS) may be employed. In neonates and young infants, this high-tech support is burdened by specific technical complexity. Despite the potential risks related to this aggressive approach, ECLS is a life-saving procedure in 65% of pediatric viral pneumonia and in 73% of sepsis cases. Here, we report the successful outcome of a 51-day formerly preterm infant, suffering from a surgical necrotizing enterocolitis (NEC), complicated with hospital-acquired pneumonia due to influenza A (H1N1) virus. She developed a severe respiratory failure, unresponsive to conventional therapy, and successfully treated with ECLS. To our knowledge, this is the first report on the use of ECLS in a formerly preterm infant, suffering from NEC complicated by influenza A (H1N1) virus infection.

## 1. Introduction

Worldwide, seasonal influenza represents a major health issue, affecting each year millions of people across all ages groups, causing substantial morbidity and mortality [1]. In the pediatric respiratory disease scenario, influenza has an established role, especially that caused by influenza A (H1N1) virus, which has been associated with severe illness in the youngest population since the virus emerged during the 2009 pandemic [2]. Neonates and infants less than 6 months of age are known to be at high-risk for complications of influenza A, most of them requiring hospitalization [3]. The presence of basal complex prematurity-related comorbidities (i.e., necrotizing enterocolitis, bronchopulmonary dysplasia, etc.) makes those patients even more prone to developing acute respiratory and cardiocirculatory deterioration in case of an intervening superinfection. Despite controversies [4,5], early diagnosis, timely administration of appropriate antiviral drugs and a proper supportive therapy provide a better clinical outcome in hospitalized patients [6,7]. Nevertheless, influenza illness, especially in the most vulnerable patients, can cause serious (cardio)-respiratory failure requiring aggressive support to allow healing and improve survival [3,7].

In the present study, we report on a former preterm infant, suffering from a surgically managed NEC, complicated with hospital-acquired pneumonia due to influenza A (H1N1) virus. As she developed severe respiratory failure, unresponsive to conventional therapy, she was successfully treated with extracorporeal life support (ECLS). 

## 2. Case Report

A 51-day female infant, formerly preterm, hospitalized in our neonatal intensive care unit (NICU) since birth, presented at 40 weeks of post-menstrual age (PMA) with respiratory distress complicating post-operative abdominal course. She was born in January 2016 at 32 weeks of gestational age by caesarean section for preterm labor after an uncomplicated pregnancy, with a birth weight of 1955 g (50th–75th percentile) and Apgar score 4/8/8 at 1/5 and 10 min, respectively.

At birth, she developed a moderate respiratory distress syndrome, for which she required endotracheal intubation, conventional mechanical ventilation (CMV) for 2 days, surfactant therapy, and subsequently non-invasive ventilation (NIV) for 5 days. On day of life (DOL) 10, she presented a stage II necrotizing enterocolitis (NEC), according to Bell’s criteria, managed conservatively with bowel rest, parenteral nutrition, and intravenous antibiotics. On DOL 45, colonic stenosis, as a late complication of NEC, required partial surgical resection of the left colon. Following an early extubation at 36 h, the post-operative course was complicated after 48 h by bowel evisceration, requiring re-intubation and emergency second surgery. 

On DOL 50, her general conditions worsened, she gradually became sick-looking, poorly perfused, febrile, tachycardic, tachypnoeic with increased oxygen requirements and work of breathing in CMV. As she presented with a sepsis-like illness, she underwent an extensive diagnostic workup. Laboratory tests revealed normal plasma levels of white blood cells (10,030/mm^3^) and neutrophils (7050/mm^3^), and elevated serum level of C-reactive protein (6 mg/dL, normal value < 0.5 mg/dL). A blood culture was performed and, pending results, empiric therapy with meropenem and vancomycin was started intravenously. 

On DOL 51, a multiplex respiratory virus panel polymerase chain reaction (multiplex PCR) assay performed on nasopharyngeal swabs and tracheal specimens turned out to be positive for influenza A (H1N1) virus. Antiviral therapy with oseltamivir (3 mg/kg/dose twice daily orally) was immediately started and standard isolation measures were undertaken, to limit nosocomial transmission. The patient was moved to the isolation room, and nursing staff cohorting was predisposed. As H1N1 case was identified in the NICU, all exposed neonates were tested, cohorted and given oseltamivir prophylaxis (3 mg/kg/day for 10 days). None of them tested positive for influenza A. Of note, the patient’s father had experienced uncomplicated flu-like symptoms in the previous week. 

A chest X-ray at the onset of respiratory symptoms showed diffuse, bilateral, interstitial lung infiltrates, consistent with viral pneumonia. The patient received CMV for 48 h, then switched to high-frequency oscillatory ventilation with no significant clinical or radiologic improvement. A trial with inhalatory nitric oxide was made with no beneficial effect. Dopamine (5 µg/kg/min) and hydrocortisone (0.1 mg/kg/h) were required for hemodynamic support. Serial chest radiographs showed worsening bilateral patchy infiltrates involving both lungs with air bronchograms and no air leaks.

Respiratory status deteriorated despite maximal conventional critical care support. By DOL 53, oxygenation index (OI) was greater than 20 and increased up to 40 in 24 h. OI values (>20 for more than 24 h, >40 for more than 4 h), persistent hypoxemia, mixed acidosis (low pH, hypercapnia, lactate >3 mmol/L) and hypotension were all criteria for extracorporeal life support (ECLS). Heart ultrasound examination before cannulation showed normal contractility, with no signs of pulmonary hypertension. Based on baseline characteristics (abdominal surgery, fluid overload) and potential cannulation difficulties (small vessel vs. double lumen VV cannula size), ECLS was the modality of choice, provided there were no contraindications (Table 1).

**Figure 1:**
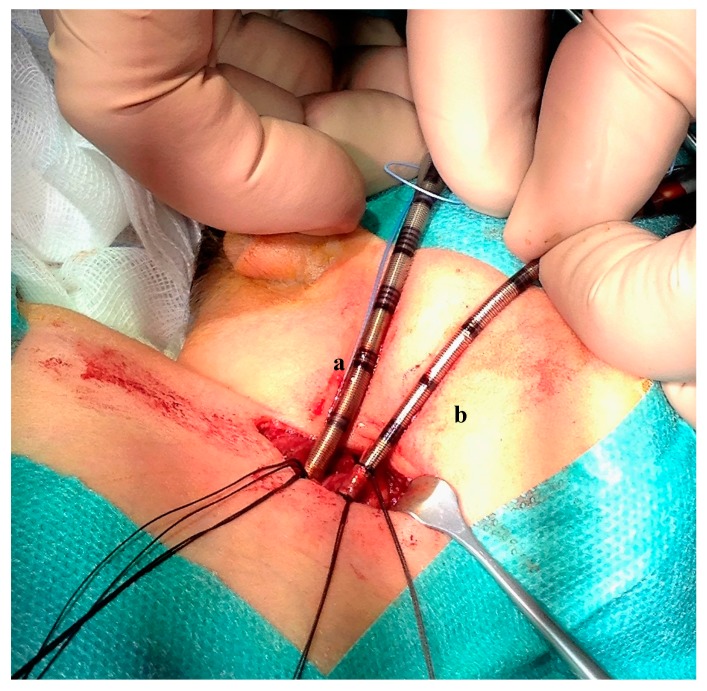
Peripheral extracorporeal life support. Surgical cannulation site in the neck: drainage cannula (10 French) inserted in the jugular vein (**a**), inflow cannula (8 French) inserted in the carotid artery (**b**).

**Figure 2:**
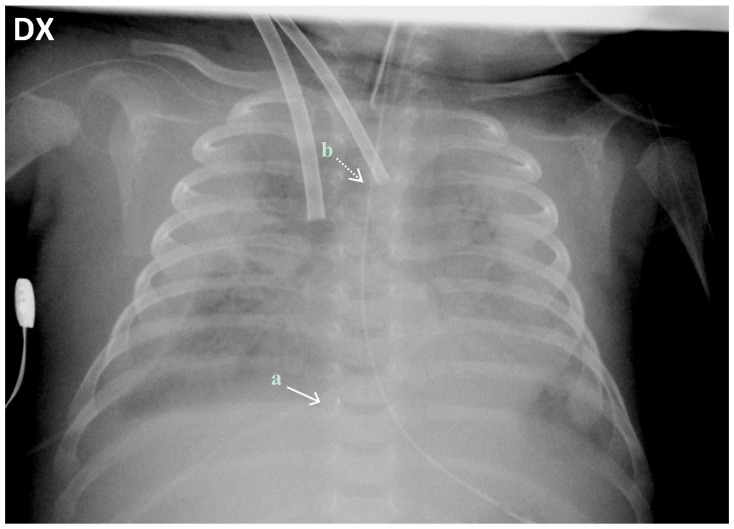
Chest X-ray of the patient 48 h after the start of ECLS support. Correct positioning of the (**a**) distal tip of the drainage cannula in the junction between the superior vena cava and the right atrium (straight white arrow) and (**b**) distal tip of the inflow cannula in the junction between the right common carotid artery and the aortic arch (dotted white arrow).

The patient weighed 3300 g at the beginning of the procedure. Surgical open cannulation was performed via the neck vessels, through a surgical exploration and dissection of the vessels (Figure 1). Correct cannula position was confirmed by chest X-ray (Figure 2) and trans-thoracic echo. Cardiopulmonary bypass (Quadrox iD, Maquet Getinge^®^) was established from the right internal jugular vein (10 Fr Medtronic^®^) to the right carotid artery (8 Fr Medtronic^®^), maintaining a blood flow of 350–500 mL/min (Cardiac Index 120–150 mL/kg/min).

Soon after the start, hemodynamic status rapidly improved and inotropic support was stopped. Mechanical conventional ventilation was initially set to “rest setting” to minimize ventilator-induced lung injury (PIP 20, PEEP 10, RR 15, FiO_2_ 0.21), and later adjusted to guarantee adequate lung recruitment (Table 1). Slow continuous ultrafiltration was required for severe fluid overload, combined with furosemide administrations and fenoldopam continuous infusion to increase urine output. 

Dexamethasone and surfactant lavages were applied with a gradual improvement in the native lung compliance. Serial blood cultures turned out to be negative. Antibiotic therapy was continued for a total of 14 days. Swabs tested repeatedly positive for influenza A (H1N1) virus on three samples (DOL 51/58/61), with a maximal viral load of 7700 copies/mL on ECLS day 9 (DOL 61). The high viral load still present after 10 days of antiviral therapy led us to perform a sequence analysis of the H1N1 strain infecting our patient, in order to investigate a possible resistance to oseltamivir, which was not confirmed. Neuraminidase (NA) and hemagglutinin (HA) sequence analysis showed no known mutation conferring resistance to oseltamivir. Antiviral therapy was continued up to 18 days, with a slow but effective clearance of the virus. No drug-related adverse event was observed.

**Figure 3:**
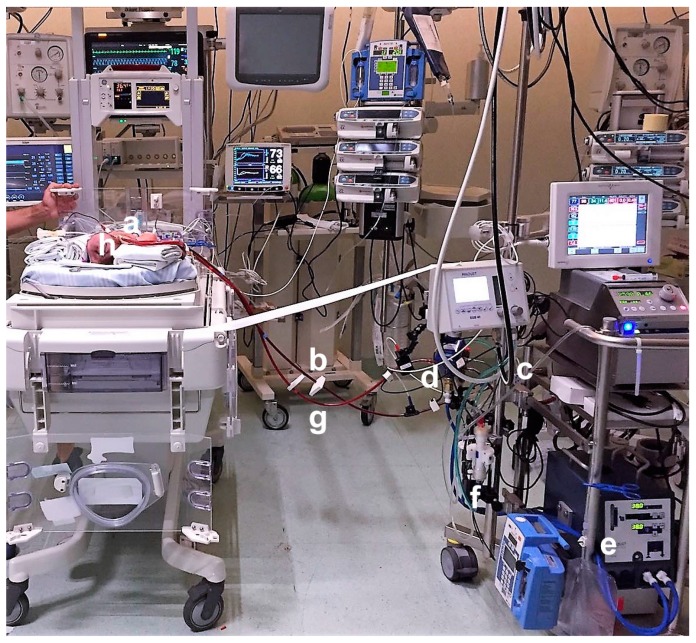
The ECLS circuit includes (**a**) a drainage cannula connected to (**b**) the outflow line. The blood is drained by (**c**) a non-occlusive centrifugal pump and is injected through (**d**) the membrane oxygenator to be oxygenated, decarboxylated and warmed through a heater (**e**); The hemofilter is in line (**f**) with the circuit. The blood is reinfused to the patient by (**g**) the inflow line and (**h**) the inflow cannula.

ECLS support was provided for a total of 13 days (Figure 3); the main circuit-related complication was a disseminated intravascular coagulation (DIC), which required a change of the ECLS circuit on day 9 of support. Heparin was modulated to maintain target ranges of ACT (180 and 220 s), aPTT (1.5–2.5 ratio) and antiXa (0.3–0.7 U/mL). Thromboelastography was employed as an adjunctive point of care coagulative test, performed four times a day or after heparin dose changes, in association with blood tests. Antithrombin (AT) was supplemented to reach adequate plasma levels (AT > 80%) and platelets (PLT) were normally transfused in case of count below the limit (PLT < 80,000 mm^3^). On day 4 the serial inspections of the circuit revealed the presence of a small clot (<1 cm) on the venous side of the oxygenator. The suspicion of circuit-related DIC was raised, when D-dimers levels kept rising in the following days, combined with a prolonged prothrombin time, hypofibrinogenemia and thrombocytopenia. The sepsis/SIRS in a surgical patient on extracorporeal circuit was a reasonable predisposing underlying factor, while the clot in the circuit was probably the trigger for the activation of coagulation. Prompt therapy with plasma was started and elective circuit change was predisposed, with an immediate improvement of coagulative tests. Clinically, the patient did not experience any bleeding or thrombosis.

Close neurological monitoring, carried out through continuous near infrared spectroscopy (INVOS^®^), cerebral function monitoring (CFM) and serial cerebral ultrasound scans, was normal throughout the procedure. 

As soon as the respiratory status (compliance, tidal volume), the hemodynamics and the overall general clinical conditions improved, we adopted a “slow weaning” strategy, reducing blood flow by 20 mL/kg/h when allowed by the stability of vital parameters (SpO_2_ > 90%, PAM > 45 mmHg, SvO_2_ > 70%). The weaning process, carried out over 24 h with a parallel adequacy of sweep gas and anticoagulation therapy, was uneventful. Once the patient was ready for native lung use, she was surgically decannulated and successfully disconnected from ECLS. 

She required 2 additional days of CMV and 20 days of NIV. A brain magnetic resonance imaging performed on DOL 88 (PMA 45 weeks) was normal for age. At 2 months corrected age (DOL 90) the patient was discharged with no medications and no supplemental oxygen. Clinical follow-up assessments revealed adequate development with no respiratory or neurologic impairment (assessed by a 12-month Bayley-III scale) at one year of age.

## 3. Discussion

In this study, we describe the clinical features and the treatment of a 51-day-old female, formerly preterm, suffering from a surgical perinatal NEC, whose course was complicated by severe pneumonia due to influenza A (H1N1) virus. NEC is one of the most impactful complications of prematurity, in terms of outcome [8]. The viral infection, acquired in March 2016, led to a general deterioration with a refractory respiratory failure, successfully managed with ECLS. 

In 2016, in the United States, influenza activity was reported to be lower, to peak later and to last longer, if compared to previous years [9]. Influenza A (H1N1) was the most frequently reported subtype [9]. The hospitalization rate for all ages was 31.3 per 100,000 people, and the number of influenza-related deaths peaked at 7.9% in March, lower than previous influenza seasons [2].

Infants and young children are more prone to developing severe illness and influenza-related complications, requiring hospitalization up to intensive care [3]. Those critical children most frequently have known comorbidities, such as bronchopulmonary dysplasia [10]. Severe pneumonia leading to respiratory failure and refractory hypoxemia may require ECMO support [11,12,13]. In case of baseline comorbidities or impending cardiovascular failure, ECLS may be a reasonable life-saving option [11,12,13]. 

In a recent retrospective study, in 73 infants under 6 months of age with influenza A virus infection, pneumonia was reported to be 4%, hypoxemia 25%, and death occurred in 3% of cases. In only one case was ECMO required, but the patient died of intracerebral hemorrhage [3].

Although survival rates in reversible pulmonary conditions and sepsis have increased with extracorporeal support, substantial and serious complications may result from its use [11]. The management of a neonate or a young infant on ECMO/ECLS may be particularly challenging at times. Besides technical issues (cannulation, circuit design, hemodilution), organ immaturity may add a specific risk factor for injuries [13]. Brain lesions are the major complications of this highly invasive approach in this age group [14]. The ELSO registry collects data on patients treated at over 400 international centers. According to its latest report, the prevalence of cerebral infarction and hemorrhage is 6.9% and 7.5%, with a survival rate decreased to 53% and 43%, respectively [15]. In general, hemostatic disorders remain the leading causes of ECLS-related morbidity and mortality, across all age-groups and organ involvement [16]. Renal function may be compromised in two-thirds of infants on ECLS, of which about 11% progresses to organ failure requiring replacement therapy [17]. As acute kidney injury in childhood may predispose an individual to chronic kidney disease in adulthood, efforts to prevent it should be made, together with a close multidisciplinary follow-up [18]. Nevertheless, although its complexity and related risks, ECLS is still a viable option in selected cases, such as life-threatening conditions. They may be related, as in this case, to a trigger (superinfection) which contributed to further deteriorate an already complex basal situation (systemic inflammatory response syndrome (SIRS)/sepsis/fluid overload/abdominal surgery). The survival rate of children supported with respiratory ECMO for a viral pneumonia is estimated to be around 65% and for sepsis 73% [15,19]. 

The case we presented highlights possible life-threatening conditions related to influenza A (H1N1) virus infection and prematurity comorbidities (previous mechanical ventilation, surgically managed perinatal NEC). Using this brief report, we provide data to support, in selected cases, aggressive management with ECLS procedure.

Our patient acquired influenza A (H1N1) virus infection during hospitalization in NICU, probably from her father, in a critical period following an emergency abdominal surgery. Based on patient characteristics, we decided on a venous-arterial (VA) bypass, given the imminent risk of cardiovascular deterioration. Hypotension and high metabolic rate are common features in septic patients, and in these cases, oxygen delivery achievable with VV-ECMO may be inadequate. Moreover, in the neonate/young infant, the only cannulation option is limited to the right vessel of the neck, because of well-known technical issues related to the size of the cannulas. Therefore it is clear how, in this specific population, a later conversion from VV-ECMO to ECLS is not easily achievable. To us, these concepts justify the choice of VA support, since the start, in a neonate with a sepsis/SIRS-like clinical features and surgical comorbidities. The viral pneumonia was an additive factor, which contributed to the general clinical deterioration. Although H1N1 virus is relatively uncommon in NICUs, neonatologists should be aware of this possibility and be proactive in preventing it [20,21]. Given that neonates and young infants with influenza often have a sepsis-like illness, a complete diagnostic workup should be done especially during the epidemic season [21]. 

Once a case of influenza A (H1N1) occurs in NICU, healthcare workers should be ready to cope with the infection, promptly treating the patient and, concurrently, preventing an epidemic spread of the virus in the NICU [20]. Implementation of infection control measures has a crucial role in reducing the impact of influenza, and this is especially valuable when it comes to delicate patients, such as a young infant with underlying known medical conditions as prematurity, bronchopulmonary dysplasia, surgical or cardiopulmonary diseases and neurological impairment [10]. Influenza vaccine and reinforced hygiene measures should be strongly recommended for healthcare workers and parents of preterm neonates, chronic patients with comorbidities during the epidemic season [6,7,20]. 

Once influenza A (H1N1) virus infection is contracted in high-risk patients, prompt treatment (preferably within 48 h of symptoms onset) with oseltamivir should be started, as recommended by AAP, CDC and Infectious Diseases Society of America (IDSA) [6,7,20]. Although in 2014 the efficacy of neuraminidase inhibitors (NAIs) for influenza has been questioned by the Cochrane review [4], the AAP has confirmed in the same year the potential benefit of antiviral drugs [6]. To reinforce this concept, AAP pointed out how the Cochrane analytic approach itself (data from healthy outcome patients with a mild illness) might have underestimated the therapeutic value of NAIs against influenza, especially in high-risk hospitalized patients [6]. In response to 2009 H1N1 pandemic, oseltamivir was approved under an Emergency Use Authorization (EUA) for treatment and chemoprophylaxis in children less than one-year-old [22]. The drug is generally well tolerated in neonates and young infants [3]. If the patient’s clinical conditions do not improve, despite adequate treatment, clinical suspicion of oseltamivir drug resistance should rise [23,24]. In our patient, PCR was extremely useful to monitor viral clearance and to define the duration of antiviral treatment. In addition, using PCR, we verified that none of the neonates or young infants who received oseltamivir for antiviral chemoprophylaxis developed influenza, and resistance to oseltamivir in the affected patient was not demonstrated. 

To our knowledge, this is the first report on the successful use of ECLS in a young infant, formerly preterm, suffering from complex baseline comorbidities (surgically managed perinatal NEC, SIRS), further complicated by Influenza A (H1N1) virus infection.

## 4. Conclusions

Preterm birth may lead to complex comorbidities, among which NEC plays an important role in the outcome. Influenza A (H1N1) virus infection as well can hesitate in significant morbidity and mortality, especially in comorbid neonates and young infants who can acquire the infection during their hospital stay. Preventive strategies, such as healthcare workers and parental vaccination, as well as implementation of infection control practices during the epidemic season, are crucial to protect the vulnerable population hospitalized in NICU. In case of severe respiratory failure, unresponsive to maximal conventional therapy, occurring in delicate patients at risk for hemodynamic instability, ECLS is indicated and may be life-saving. 

## Figures and Tables

**Table 1:** Main respiratory and hemodynamic settings before, during and after extracorporeal life support (ECLS) support.

ECLS Day	−2	−1	0	1	2	3	4	5	6	7	8	9	10	11	12	13	+1	+3
HR mean (range)	175 (155–185)		135 (120–170)														120 (110–145)	
SpO_2_ mean (range)	95	90–92	97 (93–100)														98 (96–100)	
AP mean (range)	30 (25–35)		55 (45–90)														45 (40–55)	
SvO_2_ mean (range)	NA		75 (72–80)														NA	
pH (median ± SD)	7.24 ± 0.12		7.4 ± 0.04;														7.4 ± 0.06	
pO_2_ (median ± SD)	40 ± 9.25		65 ± 11.86														63 ± 8.54	
pCO_2_ (median ± SD)	72 ± 15.23		47.4 ± 5.71														45 ± 7.21	
Lactate (median ± SD)	3.5 ± 1.1		0.9 ± 0.4														0.8 ± 0.5	
BE (median ± SD)	−1 (±0.5)		1 ± 1.64														1.3 ± 1.2	
Temperature (median ± SD)	36.6 ± 0.1		36.5 ± 0.2														36.5 ± 0.4	
**Respiratory settings**																		
Plan	Deterioration-start ECLS			Lung Rest										ECLS Weaning			Extubation	
Mode	PC/AC	HFO	PC/AC											PC/AC			PC/AC	NIV
FiO_2_	0.8	1	1	0.3	0.3	0.3	0.3	0.3	0.3	0.4	0.5	0.3	0.3	0.3	0.3	0.3	0.3	0.28
PIP	34	NA	NA	20	25	25	25	25	25	25	25	25	25	25	25	25	25	9
PEEP	6	NA	NA	10	10–13	10	10	10	10	10	10	7–10	7–9	6–8	8	8	9	5.5
Paw	12	18	20	12	14	12	12	12	12	12	12	10	10	9	10	10	12	6.5
RR/Hz	40	8	8	15	15	15	20	20	15	15	20	25	25	25	25	25	25	25
Ti/I:E	0.36	1:2	1:2	0.4	0.4	0.6	0.4	0.4	0.6	0.6	0.6	0.6	0.4	0.4	0.4	0.4	0.4	1
Vt (mL/kg)	4	1	1.5	1.8	2.3	2.1	2.4	2.2	2.4	2.3	3.2	3.5	4	4.6	4.8	4.5	4.7	NA
Compliance C20:C	NA	NA	NA	0.7	0.7	0.8	0.8	0.6	0.8	0.8	1	1	1	1.2	1.1	1.4	1.4	NA
Dynamic Compliance (Cdyn) (mL/cmH_2_O)	NA	NA	NA	0.1	0.1	0.2	0.2	0.1	0.1	0.1	0.3	0.4	0.6	0.8	0.8	1	1	NA
OI	13 (12–15)	28 (25–35)	40 (35–48)	NA													NA	
Surfactant BAL													X					
Dexamethasone													X	X	X	X	X	
**Circulatory settings**																		
Plan	Deterioration-start ECLS										Circuit change							
LPM pre-UF	NA		0.6	0.6	0.6	0.6	0.57	0.65	0.54	0.54	0.56	0.5	0.6	0.5	0.4	0.35	NA	NA
LPM post-UF	NA		0.6	0.6	0.45	0.45	0.47	0.52	0.44	0.44	0.45	0.5	0.44	0.40	0.35	0.25	NA	NA
RPM	NA		2600	2610	2610	2610	2845	2615	2615	2795	2915	2915	2280	2220	2025	1800	NA	NA
FiO_2_	NA		0.5	0.5	0.5	0.5	0.7	0.9	0.5	0.5	0.6	0.3	0.3	0.3	0.3	0.2	NA	NA
Sweep gas	NA		0.45	0.45	0.54	0.54	0.55	0.60	0.55	0.60	0.55	0.40	0.40	0.40	0.40	0.40	NA	NA
P ven	NA		−15	−15	−15	−15	−15	−16	−29	−20	−30	−30	−15	−15	−15	−15	NA	NA
P int	NA		180	180	180	180	180	169	190	170	174	180	180	180	180	180	NA	NA
P art	NA		165	165	165	165	165	155	175	156	160	165	165	166	166	165	NA	NA
Delta P	NA		15	15	15	15	15	14	15	14	14	15	15	14	14	15	NA	NA
Dopamine (µg/kg/min)	5	10	10	stop	no													
Hydrocortisone (mg/kg/h)	no	0.1	0.2	stop	no													
**Fluid management**																		
Fluid overload (%) *	+3	+0.5	0	+0.5	+1.5	+1	−0.5	−1.5	0	−2.5	+1	0	+0.5	+0.3	0	−1	−1	−1.5
Weight (g)	Entry weight: 3310 g		NA														Weight post ECLS: 3420 g	
Urine output (cc/kg/h)	2	1.5	2.5	3	6	4.5	5.7	3.9	5	4	5	4.5	4.2	7.3	5	4.5	3.5	3.5
Ultrafiltration	NA	NA	off	on												off	NA	NA
Furosemide (mg/kg/24 h)	no			4					3			2				no		
Fenoldopam (μg/kg/min)	no		0.2													no		
Albumine (g/kg/24 h)	no		0.5				no		0.5				no					
Nitroprussiate (μg/kg/min)	no													0.1–0.3		stop		

* Table 1 Trend of vital parameters, gas analysis main values, respiratory and circulatory settings before ECLS (day −2, −1), during ECLS (days 0–13) and after ECLS discontinuation (day +1, +3). ECLS: extracorporeal life support; SD: standard deviation; HR: heart rate; SpO_2_: peripheral arterial oxygen saturation; AP: arterial pressure; SvO_2_: central venous oxygen saturation; BE: base excess; pO_2_: partial pressure of oxygen; pCO_2_: partial pressure of carbon dioxide; FiO_2_: fraction of inspired oxygen; PC/AC: pressure controlled/assisted controlled; HFO: high frequency oscillatory ventilation; NIV non-invasive ventilation; PIP: peak pressure; PEEP: positive end-expiratory pressure; Paw: mean airway pressure; RR: respiratory rate; Hz: hertz; Ti: inspiratory time; I:E: inspiration/expiration ratio; Vt: tidal volume; OI: Oxygenation index; BAL: bronchoalveolar lavage; LPM: liters per minute; UF: ultrafiltration; RPM: revolutions per minute; Fluid overload (%) = [(fluids IN–fluids OUT)/entry weight ] × 100; h: hours.
